# COVID-19: THE NEED FOR INCREASED RECOGNITION OF CUTANEOUS MANIFESTATIONS – LETTER TO THE EDITOR

**DOI:** 10.21010/Ajidv17i2S.5

**Published:** 2023-08-01

**Authors:** Odekunle A, Kafi O, Olaiya A, Olatunji O

**Affiliations:** 1 Department of Medicine, East Lancashire Hospitals NHS Trust, Haslingden Rd, Blackburn, BB2 3HH, England; 2 Department of Medicine, University Hospital of Coventry and Warwickshire, Clifford Bridge Rd, Coventry, CV2 2DX, England; 3 Department of Medicine, Newcastle Hospitals NHS Foundation Trust, Queen Victoria Road, Newcastle upon Tyne NE1 4LP, England; 4 Department of Medicine, South Tynesideand Sunderland NHS Foundation Trust,Kayll Road, Sunderland, Tyne &Wear,SR4 7TP, England

**Keywords:** Covid-19, Skin, Cutaneous Manifestations

## Introduction:

It has been over three years since the first case of covid-19 was discovered in Wuhan, China. Since then, there have been a lot of discussions around symptoms and sequelae of covid-19 such as respiratory, cardiac, and neurology problems. However, not a lot of attention has been drawn to skin manifestations of this disease despite the fact that the prevalence of covid-19 associated skin lesions could be as high as 20% in some countries (Tan *et al.*, 2021).

## Discussion:

Skin conditions associated with covid-19 are quite diverse; however, chilblains have been the most commonly reported skin manifestation in many studies (Jamshidi *et al.*, 2021; Klejtman, 2020; Perna *et al.*, 2021; Tan *et al.*, 2021). These lesions which are commonly found on the hands, feet, and trunk could also include livedo reticularis, purpuras, macular or maculopapular exanthem, vesicles, and cold urticaria. It is important to recognize these skin manifestations as sometimes they could precede the systemic manifestations of covid (Klejtman, 2020; Jamshidi *et al.*, 2021). This is even more important as the protocols for covid-19 screening are being de-escalated in many countries; presentation with skin manifestations if recognized, could be an indication for testing an individual for covid-19. For example in England, patients are no longer routinely tested for covid-19 on admission to the hospital (NHS England » COVID-19 Testing Policy Update – Changes to NHS Use Cases, n.d.).

Furthermore, while many of these skin manifestations are self-limiting, some may require specialist treatment (Jamshidi *et al.*, 2021; Perna *et al.*, 2021), delay in diagnosis as covid-19 related skin disease could result in a delay in referral to dermatologists for appropriate treatment. With about 70% of those with skin lesions vasculitis and thrombosis discovered, it is crucial to not miss these cutaneous manifestations (Perna *et al.*, 2021).

## Conclusion:

Finally, it is important to create more awareness about the skin manifestations of covid-19 especially among the front-door health workers in the emergency department and acute medical units so as to ensure increased recognition of the condition to ensure patients get prompt and adequate care.

### Conflict of Interest:

The Authors declared that there is no conflict of interest associated with this study.
